# Evaluating the parent–adolescent communication toolkit: Usability and preliminary content effectiveness of an online intervention

**DOI:** 10.1002/nop2.107

**Published:** 2017-11-30

**Authors:** Elaine Toombs, Anita Unruh, Patrick McGrath

**Affiliations:** ^1^ Department of Psychology Lakehead University Thunder Bay ON Canada; ^2^ Centre for Family Health IWK Health Centre Halifax NS Canada

**Keywords:** Health, parent intervention, parent training, parent–adolescent communication, parenting, web‐based intervention

## Abstract

**Aim:**

This study aimed to assess the Parent–Adolescent Communication Toolkit, an online intervention designed to help improve parent communication with their adolescents. Participant preferences for two module delivery systems (sequential and unrestricted module access) were identified.

**Design:**

Usability assessment of the PACT intervention was completed using pre‐test and posttest comparisons. Usability data, including participant completion and satisfaction ratings were examined.

**Methods:**

Parents (*N *=* *18) of adolescents were randomized to a sequential or unrestricted chapter access group. Parent participants completed pre‐test measures, the PACT intervention and posttest measures. Participants provided feedback for the intervention to improve modules and provided usability ratings. Adolescent pre‐ and posttest ratings were evaluated.

**Results:**

Usability ratings were high and parent feedback was positive. The sequential module access groups rated the intervention content higher and completed more content than the unrestricted chapter access group, indicating support for the sequential access design. Parent mean posttest communication scores were significantly higher (*p *<* *.05) than pre‐test scores. No significant differences were detected for adolescent participants. Findings suggest that the Parent–Adolescent Communication Toolkit has potential to improve parent–adolescent communication but further effectiveness assessment is required.

## 1 INTRODUCTION

Parent–adolescent conflict is common (Smetana, 2011). Most conflicts that occur within the parent–adolescent relationship are minor, but they differ from parenting of school‐aged children (Ralph et al., 2003). These common adolescent parenting issues include challenging of authority, increasing personal independence, sibling disagreements and negotiating new responsibilities (Laursen & Collins, 2009; Smetana, 2011). To resolve these conflicts, parenting practices may require modification, specifically within parent–adolescent communication. Poor parent–adolescent communication is associated with detrimental parent and adolescent health outcomes and can impact the choices an adolescent makes (Resnick et al., 1997).

Increased and escalating conflict can impair the quality of the parent–adolescent relationship (Smetana, 2011). The strength of the parent–adolescent relationship can influence adolescent decisions regarding sex (Wilson & Donenberg, 2004), education (Hill et al., 2004), alcohol use (Chaplin et al., 2012) and tobacco use (Tilson, McBride, Lipkus, & Catalano, 2004). Positive parenting results in higher self‐esteem, higher academic achievement and better emotional adjustment for adolescents (Vasquez, Patall, Fong, Corrigan, & Pine, 2016). A strong relationship between a parent and adolescent can protect adolescents from emotional distress, suicidal thoughts and violence (Resnick et al., 1997).

Adolescence can be a challenging developmental stage to parent (Larson, Richards, Moneta, Holmbeck, & Duckett, 1996). Increased conflict within a parent–adolescent relationship is associated with higher levels of parental stress (Pasley & Gecas, 1984). Parents have rate adolescence as the most difficult stage of parenting (Anderson, 2008). Parents of adolescents report lower levels of emotional functioning, less competence, lower self‐esteem and less life satisfaction, compared to parents of younger children (Larson et al., 1996). Additional factors increase parental stress such as adolescent mental health concerns, parent health status, family poverty or lack of parenting supports (Anderson, 2008). These issues can reduce the use of effective parenting techniques, reduce parent–adolescent conflict resolution, and increase the likelihood of detrimental adolescent outcomes (Smetana, 2011).

Despite increased stress within parent–adolescent relationships, there are few accessible supports available to meet the needs of these parents. Effective parent interventions to improve parent–adolescent relationships, reduce parental stress and improve parent–adolescent communication are required. Parenting training programs can be difficult to obtain for many families due to location of the service, cost and waiting lists (McGrath, Lingley‐Pottie, Emberly, Thurston, & McLean, 2009; Reid & Brown, 2008). Parents are often discouraged by the treatment options available to them (Shanley, Reid, & Evans, 2008).

Available behavioural change parent training programs include Adolescent ParentWays (Taylor et al., 2015), Triple P (Nowak & Heinrichs, 2008) and Parenting Adolescents Wisely (Kacir & Gordon, 2000). Taylor et al. (2015) have argued that despite the effectiveness of evidence‐based programs, further parenting solutions that are more accessible, affordable and relevant, are required. Increasing options of care and accessibility of parent training programs will lead to better outcomes for families.

### 1.1 The parent–adolescent communication toolkit

The Parent–Adolescent Communication Toolkit (PACT) is an online intervention targeted at parents of adolescents to improve the communication of parents with their adolescents, and their relationship. The PACT intervention (Toombs, Unruh, & McGrath, 2013) was developed with close collaboration between parents of adolescents and the research team. PACT takes principles from Gottman's couple relationship intervention (Declaire & Gottman, 2001; Gottman & Ryan, 2005; Gottman & Silver, 2015) and combines these with the Strongest Families model of care (McGrath et al., 2009, 2011) to create a strategy for parents to improve communication with their adolescents. Table 1 briefly describes each module's content.

**Table 1:** Summary of PACT module content

Section	Module title	Description
Introduction		Outlines the program and the major features.
Assessment	Taking the parent–adolescent communication test	How to determine where your conversations go wrong.
Building relationships		
Module 1:	Pay attention	How to react positively to your adolescent's attempts at emotional connection.
Module 2:	Give affection and respect	Expressing good feelings about your adolescent through compliments, praise and positive observations.
Module 3:	Create shared meaning	Finding shared Creates greater stability in relationships, allows pursuit of goals together.Create rituals to connect, have symbolic and emotional meaning.
Module 4:	Give goals room to grow	Recognize and honour the dreams and feelings within your adolescent.
Module 5:	Accept your teen and his/her influence	Be open to persuasion from your adolescent without giving in.
Module 6:	Accept one another	Accept your adolescent for who he or she is, not the person you want him/her to be
Positive communication		
Module 7:	Compromise	Avoid gridlocking by working out a decision that both you and your adolescent can agree upon and be happy with.
Module 8:	Start softly	Learn to start talking about a complaint without criticizing or insulting.
Module 9:	Repair your communication	Deescalate negative feelings during a difficult encounter with your adolescent.
Turn around negative communication (Module 10)		
	Criticism	Avoid attacking your adolescent's personality or character rather than specifics.
	Contempt	Avoid insulting and psychologically abusing your adolescent.
	Stonewalling	Avoid removing yourself from the conversation mentally.
	Defensiveness	Avoid defending yourself from insults. Nine main strategies.
	Flooding	Avoid overwhelming your adolescent with too many complaints.
Talk about difficult issues (Optional Module 11)		
		Using the intervention skills to talk to your adolescent about sex, drugs, divorce and mental health. Offers additional resources based on these.
Summary		Summarizes the program and how to solve problem.

PACT was modelled on Gottman's relationship repair strategies given the applicability of his relationship repair theories to a parent–adolescent dyad with some modifications and the high success rate of this relationship therapy. Simple communication strategies and practical suggestions are combined with activities such as writing and self‐reflection exercises for couples. Relationship repair skills can be implemented independently, without the aid of a therapist. By simplifying his research findings, Gottman provided an alternative approach to traditional face‐to‐face therapy (Gottman & Ryan, 2005). Many of Gottman's couple communication modules such as Nurturing Fondness and Admiration, Creating Shared Meaning and Turning Towards Each Other (Gottman & Silver, 2015) can be applied to a parent–adolescent dyad.

The Strongest Families empirically validated distance care model (McGrath et al., 2009, 2011) for child mental health was used as a framework for the PACT intervention. Strongest Families offers telehealth and web‐based interventions to families requiring support for childhood behavioural and anxiety disorders. Strongest Families programs implement programs for parents and children assisted by highly trained and monitored non‐professional coaches that are reachable by telephone or email. The Strongest Families program is highly effective at reducing typical treatment barriers that exist for families seeking support (McGrath et al., 2011). Programs offer evidence‐based skills that are customized to meet parent requirements. Strongest Families facilitates accessible, convenient and confidential care, in a novel and effective approach to family mental health treatment (McGrath et al., 2009, 2011).

By combining the Strongest Families model of care with Gottman's relationship repair strategies, the PACT intervention offers an alternative to traditional parenting interventions. It provides a low cost, convenient measure for parents seeking additional support for parenting their adolescents without the stigma of seeking treatment. The intervention normalizes parent–adolescent conflict, and provides specific skills to reduce these concerns. By delivering the PACT skills online, parents are able to seek information at their own pace, on their own time. PACT is designed for parents to complete without the aid of a therapist.

### 1.2 PACT delivery via Individualized Research and Intervention Software

The PACT intervention is delivered using Individualized Research and Intervention Software (IRIS) technology. IRIS software facilitates the creation of web‐based therapeutic mental health interventions in an appealing and user‐friendly format. This platform was developed by the Center for Research in Family Health research team, through a grant funded by the Canadian Institute of Health Research. IRIS is customizable and interactive for families, allowing personalized profiles and content for participants, integrating demographic information with health indicator behaviour inputs. The features IRIS offers, such as messaging services, email reminders and discussion boards, allows for customizable intervention content to suit the specific needs of the PACT intervention (Wozney et al., 2016).

To meet study demands, IRIS can modify the intervention content presentation, order and time of presentation. IRIS can deliver questionnaires, collect data and offer study completion reminders to participants. The software can track participant activity, such as time spent on each page and will track participant progress and activity by date.

### 1.3 The purpose and hypotheses of this study

The main purpose of the current study was to assess the usability of the new content and online format of PACT. We assessed the intervention usability using parent feedback to provide ideas regarding potential improvements, and alternative constructs that could better facilitate participant learning in future modifications to the PACT intervention.

PACT is a new intervention and the method of presenting the intervention content had not been assessed. The second aim of this study was to assess two different methods of intervention content delivery to yield the best participant outcomes possible. Participants were randomly assigned to either a sequential method of content delivery, meaning content must be completed in a rigid, predetermined order, or participants had unrestricted access. Participants with unrestricted module access completed modules in any order of choice independent of previous modules completed. Unrestricted module access provided more freedom but could affect how the PACT skills were learned.

Given that PACT is a novel intervention, a peripheral aim of this study was to assess outcomes of PACT for parents and adolescents' communication scores during a 6‐week study duration. Exploratory analyses examined the differences between parent–adolescent communication scores before and after completion of PACT were completed to provide preliminary information about the potential for effectiveness of the intervention in a larger study. To determine the preliminary effectiveness of PACT, parent and adolescent scores were analysed separately. Parent and adolescent depression, stress and anxiety scores were analysed using paired *t* tests. These measures were taken to determine if PACT influenced the emotional functioning of participants. In summary, the hypotheses for this study were:

- Parent usability ratings for the PACT content delivered using the IRIS platform would be high.
- Parents randomized to the sequential module access would have higher completion rates and usability ratings than those randomized to the unrestricted module access group.
- Parent and adolescent would use significantly more positive communicational strategies following the PACT intervention, as rated by the IWK‐PACC and emotional functioning (DASS) posttest scores would be significantly higher than pre‐test scores.

## 2 METHOD

**Figure 1:**
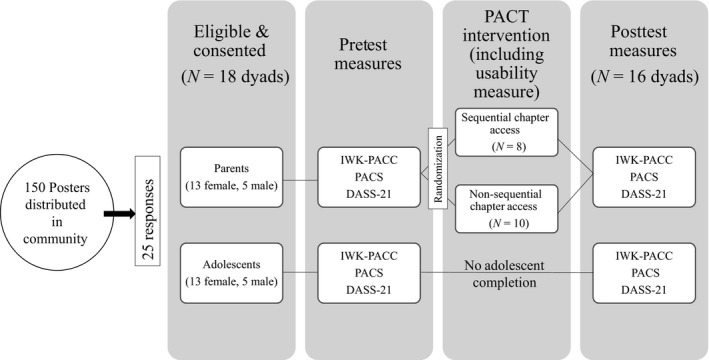
Participant flow and research design diagram

This study was completed using a pre–posttest design with a 6‐week intervention. Parent participants were randomized into two groups—sequential chapter access or unrestricted module access. Figure 1 is a diagram of the study design.

### 2.1 Study randomization

All parent participants completed identical PACT content, although how the intervention content was delivered was randomly assigned. Parents either had unrestricted module access or sequential module access, with each module unlocked only after completion of the previous module.

## 3 MATERIALS

### 3.1 Website

The study was conducted using a customized IRIS web‐based platform. All usability questionnaires and intervention content were presented using IRIS. Parent participants completed usability study portion (content presentation, data collection and preliminary descriptive analyses) entirely through the PACT website.

### 3.2 Study measures

Four questionnaires were administered. These included the IWK‐ Parent–Adolescent Communication Checklist (Unruh, Bagnell, Huguet, & Mcgrath, 2011), the Parent–Adolescent Communication Scale (PACS) (Barnes & Olson, 1985), the Depression Anxiety Stress Scale (DASS‐21) (Lovibond & Lovibond, 1995), and the Perceived Health Web Site Usability Questionnaire (PHWSUQ) (Nahm, Preece, Resnick, & Mills, 2004). The PACS is a well‐established parent–adolescent communication measure and shows high alpha reliabilities of 0.87 and 0.78, with test–retest reliabilities of 0.78 and 0.77 (Barnes & Olson, 1985). The DASS‐21 was chosen as a short measure of emotional distress with high internal consistency (Lovibond & Lovibond, 1995).

### 3.3 Participants

Eighteen parent–adolescent dyads completed the study. Adolescent participants were between the ages of 13–17 years and were a resident in their parent's home. Parent participants agreed to commit to the 6 weeks required of the study. A convenience sample of 20 dyads, recruited by word of mouth in the community, was chosen to assess the overall usability for this pilot study and for this sample to provide in depth comments on aspects of the intervention that should be modified before further implementation. Two dyads completed consent and were randomized, but did not complete any intervention content. Exploratory analyses completed from this sample were used to determine significant trends in the data in relation to the effectiveness of the intervention.

No parent had participated in another parenting or communication behavioural intervention (including any prior PACT study), or received support for a mental health problem in the previous 6 months. Parents who reported any severe psychological impairment for themselves or their adolescent were excluded from the study. All parent participants had access to the Internet. Only one parent–adolescent dyad per family could participate. Parent participants were primarily birth parents, well educated (most achieved a 2‐year college diploma or higher) and had a secondary parent in their family. No parents earned less than $20 000 per year. Parent–adolescent dyads were primarily mother‐daughter.

## 4 RESULTS

### 4.1 Hypothesis 1: Parent usability ratings of PACT delivered using the IRIS platform would be high

Participant usability module ratings were rated highly by both participant groups with Table 2 depicting the mean module ratings for the sequential access and unrestricted access participant groups. The total ratings per each of the 10 modules (obtained by averaging all participant raw scores for each chapter) were analysed using a one‐way ANOVA and did not differ significantly from one another at the *p *<* *.05 level for the ten modules [*F* (9, 98) = 0.434, *p *=* *.914)].

**Table 2:** Mean usability module ratings by participant group and total scores

Module	Sequential module access	Unrestricted module access	Total
	*M* (*SD*)	*M* (*SD*)	*M* (*SD*)
	(*N *=* *6)	(*N *=* *10)	(*N *=* *16)
Module 1	73.20 (4.82)	61.56 (5.32)	65.71 (7.62)
Module 2	71.40 (8.20)	61.80 (7.19)	65.00 (8.63)
Module 3	73.20 (6.10)	60.90 (8.85)	65.00 (9.85)
Module 4	72.2 (10.73)	59.86 (9.89)	65.00 (11.65)
Module 5	72.00 (10.10)	63.30 (9.24)	67.8 (10.14)
Module 6	69.20 (10.96)	63.00 (7.00)	66.10 (9.27)
Module 7	76.00 (1.73)	66.00 (8.03)	69.75 (8.03)
Module 8	76.33 (1.15)	65.00 (7.84)	69.25 (8.36)
Module 9	76.00 (1.73)	63.80 (9.20)	68.38 (9.44)
Module 10	77 (0.00)	64.00 (7.78)	68.88 (8.94)

#### 4.1.1 Participant usability feedback

Parents provided written feedback for each module. Few modifications were suggested. Parents found the “Relationship Memory Bank” and “Being Specific with Praise” particularly helpful. The audio‐visual of the intervention content was difficult to access on tablets and phones, and greater diversity in the family's illustrated in the videos was recommended. Parents also suggested the number of questions, the number of examples, and the repetitive content be reduced.

### 4.2 Hypothesis 2: Parents randomized to the sequential module access group would have higher completion and usability ratings than parents in the unrestricted module access group

**Figure 2:**
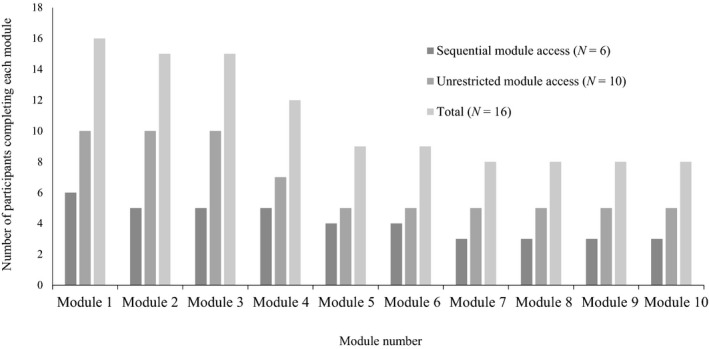
Percentage of participant PACT module completion

Two dyads did not complete the posttest study questionnaires and were removed from subsequent analyses. Remaining parent participants varied on module completion rates, with eight participants (50%) completing all ten modules of PACT. Modules of PACT are dispersed in four sections. Sixty‐two percent of participants completed the first section, and 50% completed sections two, three and four. Participation decreased by 50% after Module 4. Figure 2 depicts the percentage of parent participants who completed each module by randomization to the sequential or unrestricted module access groups.

Participant global usability ratings by each access group were analysed to determine if the sequential module access group rated the content higher than the unrestricted module access group. These global ratings were obtained by adding participant scores across modules and dividing by the total score possible for how many modules each completed. An independent *t* test detected significant differences [*t*(5) = 2.486, *p *=* *.027] between the sequential (*M *=* *0.9339, *SD *= 0.09395) and unrestricted module access groups (*M *=* *0.8056, *SD *= 0.09440) at a *p *<* *.05 level of significance.

### 4.3 Hypothesis 3: Parent and adolescent posttest communication and emotional functioning scores would be significantly higher than their pre‐test scores

#### 4.3.1 Adolescent participant outcomes

Adolescent pre‐test and posttest communication ratings did not significantly differ for both the IWK‐PACC [*t*(15) = 1.626, *p *=* *.125] and the PACS [*t*(15) = 1.478, *p *=* *.160]. Depression [*t*(15) = 1.549, *p *=* *.142] and stress [*t*(15) = 1.612, *p *=* *.128] scores did not differ. Anxiety posttest scores were significantly lower than pre‐test scores [*t*(15) = 2.394, *p *=* *.03].

#### 4.3.2 Parent participant outcomes

Paired *t* tests found posttest scores to be significantly higher than pre‐test scores for both the IWK‐PACC [*t*(15) = 2.689, *p *=* *.017] and the PACS [*t*(15) = 3.168, *p *=* *.006]. Emotional functioning for [*t*(15) = −0.194, *p *=* *.849], depression [*t*(15) = −0.831, *p *=* *.419] or stress scores [*t*(15) = −1.263, *p *=* *.226] was not significant.

Comparisons of the sequential and unrestricted chapter access groups found no significant differences. Repeated measures analysis of variance (ANOVA) tests found no significant differences between group pre‐ and posttest scores of the IWK‐PACC [*F*(2,29) = 1.736, *p *=* *.194] and the PACS [*F* (2,29) = 0.520, *p *=* *.600].

#### 4.3.3 IWK‐PACC subscale results

IWK‐PACC was divided into three primary subscales: Building Closeness and Admiration (BCA), Reducing and Repairing Conflict (RRC) and Increasing Conflict (IC). The two communication scales, BCA and RRC, were combined and the negative IC was reverse scored to produce a total IWK‐PACC communication total. Table 3 provides mean participant scores for the IWK‐PACC and the PACS.

**Table 3:** Mean Participant Pre‐test and Posttest Scores for IWK‐PACC and PACS

Measure (Max)	Parent pre‐test *M* (*SD*)	Teen pre‐test *M* (*SD*)	Parent posttest *M* (*SD*)	Teen posttest *M* (*SD*)
PACS total (50)	15.13 (9.45)	9.94 (10.07)	20.06 (9.91)	13.00 (9.90)
IWK‐PACC total (204)	111.19 (52.41)	139.50 (50.10)	133.00 (43.21)	152.82 (50.08)

The three primary subscales of the IWK‐PACC (Building Closeness and Affection, Reducing and Repairing Conflict and Increasing Conflict) were analysed using a repeated measures ANOVA to determine if significant differences exist between pre‐test and posttest scores for both parent and adolescent participants. It was found that there were no significant differences between the three IWK‐PACC subscales for either parent [*F* (2, 1) = 184.544 *p *=* *.745] or adolescent participants [*F* (2, 1) = 243.513 *p *=* *.137] at a *p *<* *.05 level of significance.

## 5 DISCUSSION

Participant usability ratings of PACT in this study were encouraging. Parents rated all of the PACT modules quite highly, thus supporting Hypothesis 1, with a small decreased rating for the audio‐visual content. Parents rated PACT as easy to use, the content to be relevant and easy to learn. All modules were rated highly. The only consistent feedback provided by parents was that the videos did not work on some devices (e.g., tablets and smart phones) and partially impaired their completion of this PACT content. More audio‐visual components were requested, depicting a greater variety of family structures, and communication situations, as videos only showed interactions within a mother–daughter dyad. Parent responses indicated that the content was somewhat repetitive. Small modifications to PACT to address this feedback are required before the next phase of testing.

Parents who were randomized into the sequential module access completed more content and rated the usability of PACT higher than participants in the unrestricted module access group. The significantly higher usability ratings by the sequential module access group provide support for this type of structure for PACT. The sequential access style of content delivery, often called tunnelling, decreases distraction of the user and ensures the user completes necessary content (Danaher, McKay, & Seeley, 2005). Due to both high usability ratings and increased benefits noted by participants, the sequential module access is the recommended style of information architecture.

Increased completion rates of the sequential module access also support the use of this design in future studies. Parents in the sequential module access group completed more of the PACT content than those randomized to the unrestricted module access group. Only two parents or 11% of participants did not complete all of the modules in comparison to a previous PACT study (unpublished data) in which six participants (30%) did not complete all modules.

Participant retention is a priority for future studies of PACT. Fifty percent of the parent participants did not continue the intervention after module 4 even though this module was rated as highly as the earlier modules. Many web‐based interventions have similar attrition rates (Eysenbach, 2005) and low completion rates reduce the opportunity to achieve strong program outcomes. More email reminders from PACT could be provided through an automated IRIS system. The attrition rate may also have been influenced by parents' perception of content repetition for modules four and five where skills are applied in more complex communication. Streamlining of content to reduce repetition will be helpful.

PACT presents foundational skills related to building a positive relationship before more challenging skills designed to reduce conflict. Parents, who had have a more positive relationship with their adolescent, may not have found the foundational skills of sufficient interest to continue to the subsequent modules. Although PACT was customized through IRIS, it is possible further personalize PACT to meet individual parent needs. IRIS uses algorithms to modify the content presentation to parents based on prior responses to questionnaires. If parents completed pre‐test assessments online, particularly the IWK‐PACC (as the IWK‐PACC directly assesses each of the PACT intervention module skills), IRIS can combine scores and determine internally the most applicable content for each parent. Further customization of content would increase participation and reduce potential boredom, frustration or dissatisfaction with skills.

Increased customization may also decrease attrition rates when combined with the sequential chapter access design due to increased relevancy and structure of the content. A systematic review by Christensen, Griffiths, and Farrer (2009), found that participation rates for randomized control trials were often much higher (ranging from 50% to 99% completion) than those for open access websites (ranging from 1% to 50%), of web interventions aimed to reduce depression symptoms. Using a sequential access design can result in better user experience, by increasing researcher control of content.

Emotional health of parents and adolescents was generally not affected by parent completion of PACT. The IWK‐PACC and PACS posttest communication measures for parents were significantly higher than pre‐test scores demonstrating that communication with their adolescent had improved. There was no significant difference in adolescents' pre‐ and posttest scores on the IWK‐PACC or the PACS likely due to the high pre‐test scores. Most adolescents in this study already had strong relationships with their parent before the intervention. Similarly, pre‐test scores on the DASS‐21 for parents and adolescents indicated low stress, anxiety and depression and these scores were not significantly changed by PACT.

Fathers tend to be less willing to participate in parenting interventions and often have a much higher attrition rate and lower satisfaction than mothers (Lee & Feldgaier, 2015). In this study, five of the 18 parents were fathers. They did not identify any gaps in the PACT content but the small sample size did not permit comparison by gender.

### 5.1 Study limitations

The main limitation of this study was the small sample size and inclusion of parent–adolescent dyads with relatively strong relationships at study outset. During the recruitment phase, six parents expressed interest in completing the research study but could not participate due to the unwillingness of their adolescent.

## 6 CONCLUSION

This study confirmed the usability of the PACT intervention and provided evidence for the best structural architecture for content delivery. Participants identified modifications to the intervention content to improve it for future use. Exploratory analyses of change in parents' perceptions of their relationship with their adolescent following PACT indicated that the PACT intervention may be a viable tool for improving parent–adolescent communication.

## ETHICAL APPROVAL

Ethical approval for this study was obtained from the Research Ethics Board at the IWK Health Centre, in Halifax, Nova Scotia, Canada.

## AUTHOR CONTRIBUTIONS

Elaine Toombs, MA, is a Clinical Psychology graduate student at Lakehead University. This project was completed as part of her thesis requirements for a Masters of Arts (Health Promotion) degree at Dalhousie University. Ms Toombs participated in study design, data collection, data analysis, manuscript drafting and revisions.

Anita Unruh, PhD, was Ms. Toombs' MA thesis supervisor. She was a Professor and Associate Dean in the Faculty of Health Professions at Dalhousie University. She contributed to all aspects of this manuscript, including study conceptualization, design, data interpretation, and initial manuscript revisions.

Patrick McGrath, OC, PhD, FRSC, FCAHS, is the Integrated Vice President Research and Innovation for Capital District Health Authority and IWK Health Centre. He is Professor of Science, Paediatrics, Psychiatry and Community Health and Epidemiology, and Canada Research Chair at Dalhousie University. Dr. McGrath sat on Ms. Toombs' thesis committee, and was involved in study conceptualization, design, data interpretation and manuscript revisions.

All the Authors have agreed on the final version and meet at least one of the following criteria [recommended by the ICMJE (http://www.icmje.org/recommendations/)]:

- substantial contribution to conception and design, acquisition of data or analysis and interpretation of data;
- drafting the article or revising it critically for important intellectual content.
